# An Approach to and Treatment of Indeterminate Biliary Strictures: A Comprehensive Review of the Literature

**DOI:** 10.3390/jcm14010029

**Published:** 2024-12-25

**Authors:** Giovanna Impellizzeri, Maria Vittoria Grassini, Giulio Donato, Claudio Giovanni De Angelis, Nico Pagano

**Affiliations:** 1 Gastroenterology Unit, Department of Oncological and Specialty Medicine, Azienda Ospedaliero-Universitaria Maggiore della Carità, 28100 Novara, Italy; g.impellizzeri@maggioreosp.novara.it (G.I.); eusdeang@hotmail.com (C.G.D.A.); 2 Section of Gastroenterology & Hepatology, Department of Health Promotion Sciences Maternal and Infant Care, Internal Medicine and Medical Specialties, PROMISE, University of Palermo, 90127 Palermo, Italy; mvittoriagrassini@gmail.com

**Keywords:** strictures, ERCP, EUS, cholangioscopy, cholangiocarcinoma

## Abstract

This review aims to focus on what we know about the management of biliary strictures of unknown etiology, especially exploring our diagnostic armamentarium in the setting of indeterminate biliary strictures. Presently, this is a current issue that has a relevant impact both on patient prognosis, often delaying diagnosis, and on overall costs associated with repeating diagnostic procedures, sometimes performed with very expensive devices. We also focus on current biliary drainage approaches, providing an overview of therapeutic options, endoscopic or not.

## 1. Introduction

Indeterminate biliary strictures still represent a diagnostic and therapeutic challenge mainly because of the lack of a clear, shared definition. This original subject often generates ambiguity in the interpretation of literature studies and hampers a universal approach. Currently, there is not a clear agreement in indeterminate biliary stricture management, even from two of the major gastroenterological societies’ guidelines. The European Society of Gastrointestinal Endoscopy *(ESGE)* guidelines [1] actually still focus on a single technique to obtain a diagnosis, depending on stricture position, whereas the latest American Society for Gastrointestinal Endoscopy *(ASGE)* ones [2] more so stress the concept that, if we have a stricture of uncertain etiology, all the available tools have to be used as soon as possible to procure a diagnosis. Today, one of the most common definitions of indeterminate stricture is a stenosis in which a diagnosis of nature is still lacking after the results of laboratory tests, cross-sectional imaging (such as ultrasonography, computed tomography (CT), magnetic resonance imaging with cholangiopancreatography (MRI/MRCP)) and endoscopic procedures, such as retrograde cholangiopancreatography (ERCP) or endoscopic ultrasonography (EUS) with standard diagnostic tools (cytology, biopsies or FNB/FNA).

Most indeterminate biliary strictures are malignant, due to cholangiocarcinoma (CCA) or pancreatic adenocarcinoma, but they also could be benign ones (iatrogenic injury, inflammatory or infectious causes, etc.) (Table 1). A quick diagnosis can lead to a better prognosis and less morbidity/mortality in both cases, one side providing an earlier curative or palliative treatment and the other avoiding useless overtreatment.

In this setting, obtaining a histological diagnosis is the key to assessing appropriate, subsequent, and patient-tailored management.

This review will focus on the many possible causes of indeterminate biliary strictures. We performed a comprehensive literature review researching the most relevant studies about this topic on the PubMed and Scopus databases using the keywords “biliary strictures”, “cholangiocarcinoma”, “ERCP-guided drainage”, “EUS-guided drainage”, and “cholangioscopy”. Starting from the different diagnostic tools, we also focus on the best therapeutic approaches to manage them.

## 2. Indeterminate Biliary Stricture Characteristics

Biliary stricture signs and symptoms are heterogeneous. Sometimes, diagnosis is accidental in patients undergoing cross-sectional imaging for other reasons. Other times, there are signs such as jaundice and fever or symptoms like abdominal pain or nausea. Clearly, a patient’s personal history plays a key role. If we have asymptomatic jaundice or weight loss, we can suspect malignancy. Conversely, if we have a history of chronic pancreatitis or of previous hepatobiliary surgery, we can suspect a benign stricture. Biliary strictures can be classified according to their location (proximal or distal to the hepatic hilum), to their growth pattern (extrinsic or intrinsic, as to say from an external compression or an intraductal growth), and to their etiology (benign or malignant). In most cases, biliary strictures are malignant: in a retrospective analysis of 342 patients with obstructive jaundice and a biliary stricture who underwent EUS/EUS + FNA, 72.5% of cases were diagnosed as malignant ones [3]. Another retrospective study evaluating EUS + FNA specifically in proximal biliary strictures identified the same proportions between benign and malignant lesions: in 71% of patients, there were findings showing adenocarcinoma [4]. Among malignant causes, we can have cholangiocarcinoma (CCA), pancreatic adenocarcinoma, hepatocellular carcinoma (HCC), gallbladder cancer, lymphomas, and metastasis.

Among the benign causes, we can have previous hepatobiliary surgery (such as cholecystectomy or liver transplant), chronic pancreatitis, chronic diseases such as primary sclerosing cholangitis (PSC) or autoimmune cholangiopathies, and infectious or ischemic causes. Despite that, today, indeterminate lesions still account for up to 20% of cases [5] after the use of first-line diagnostic tools, and so we often need further investigations.

## 3. Main Diagnostic Tools

If a biliary stricture is suspected or recognized through ultrasonography, CT, or MRI/MRCP, we need a histological sampling to define its nature. Many tests are available to reach a diagnosis, considering that none of them is always conclusive and often the final diagnosis is made by a combination of them.

### 3.1. ERCP

**Figure 1:**
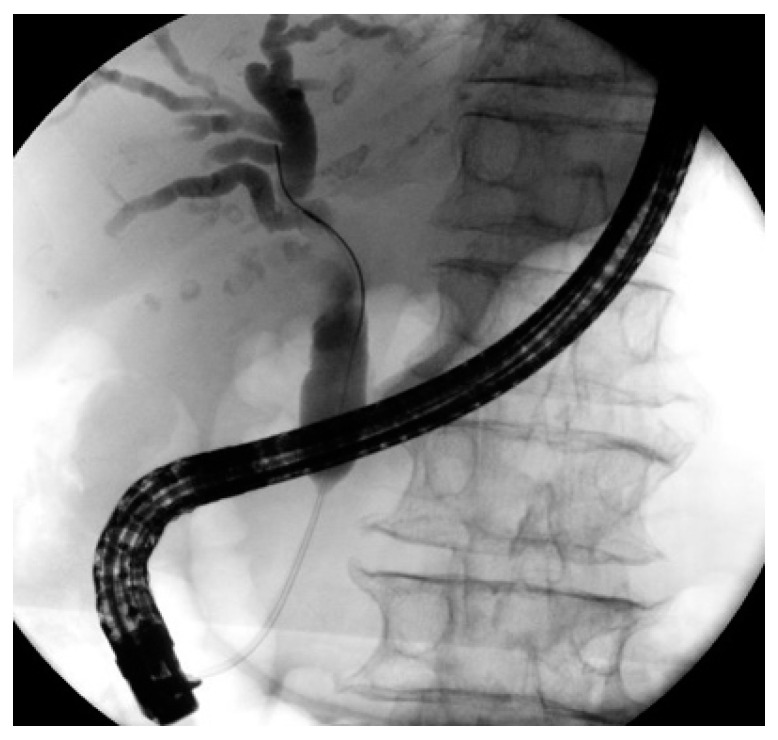
Double biliary stricture in pancreatic cancer with liver perihilar metastasis.

**Figure 2:**
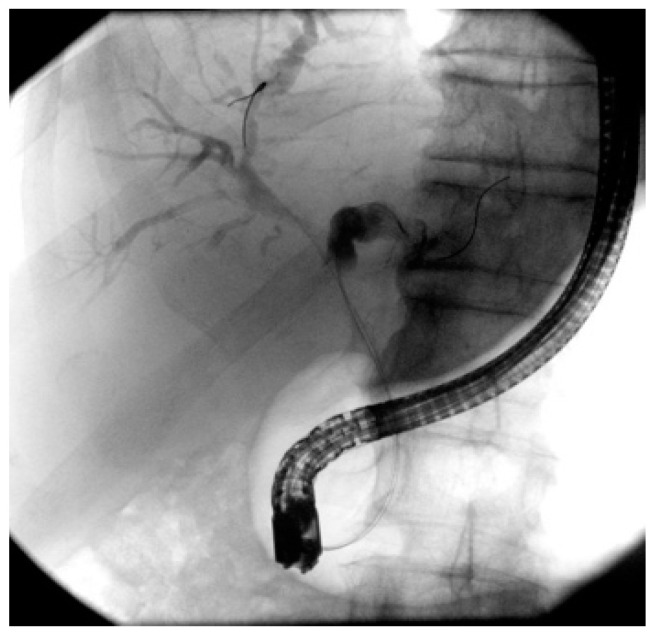
Hilar cholangiocarcinoma stricture.

ERCP has both a diagnostic and therapeutic role in this setting. During ERCP, after a successful cannulation, we perform a cholangiography that shows us the site and suggests to us a stricture nature: an abrupt narrowing is mostly typical of malignancy (Figure 1 and Figure 2) as compared to a smooth or a progressive one. ERCP is still the first-line approach technique to obtain cytological and histological samples in biliary strictures, but it is also essential in the subsequent therapeutic approach because of its palliative role. Traditionally, brush cytology, inexpensive and routinely performed, has high specificity but low sensitivity in detecting malignant biliary strictures. Intraductal biopsies, which require sphincterotomy, do not have a clear advantage over the brushing technique. A 2015 meta-analysis comparing the effectiveness of brush cytology and intraductal biopsies in biliary strictures showed that the pooled sensitivity of the brushing technique and intraductal biopsies were, respectively, 45% and 48.1%. Combining both modalities, the sensitivity modestly increased (59.4%) [6]. Brushing also allows us to obtain material for FISH (fluorescence in situ hybridization). FISH is a cytogenic technique that permits abnormality identification in a specific DNA sequence, and it was demonstrated that adding it to standard cytology analysis improves diagnostic chances. A single-center retrospective study showed that the dual-modality sampling (transductal biopsies + brushing cytology or brushing cytology + FISH) or the trimodality one (transductal biopsies + brushing cytology + FISH) performed better than brushing cytology alone in detecting malignancy, respectively, by 58.7% and 40.4% in the dual-modality samplings and by 68.3% in the trimodality one versus by 17.3% in brushing cytology alone [7]. Furthermore, another retrospective study including 168 patients with suspicious malignant biliary strictures who underwent double-tissue sampling (DTS) or triple-tissue sampling (TTS) via ERCP, showed a diagnostic sensitivity for cancer significantly higher in the TTS group than the DTS group (85.0% vs. 64.9%, respectively). Remarkably, regarding cancer type (cholangiocarcinoma vs. non-cholangiocarcinoma), the diagnostic sensitivity was higher for cholangiocarcinoma in the TTS group than in the DTS group (100% vs. 69.4%, respectively; *p* < 0.001) but not for the non-cholangiocarcinoma patients (57.1% vs. 57.1%, respectively). Thus, triple-tissue sampling can offer relevant diagnostic accuracy in suspicious biliary strictures, especially due to cholangiocarcinoma [8].

### 3.2. EUS

**Figure 3:**
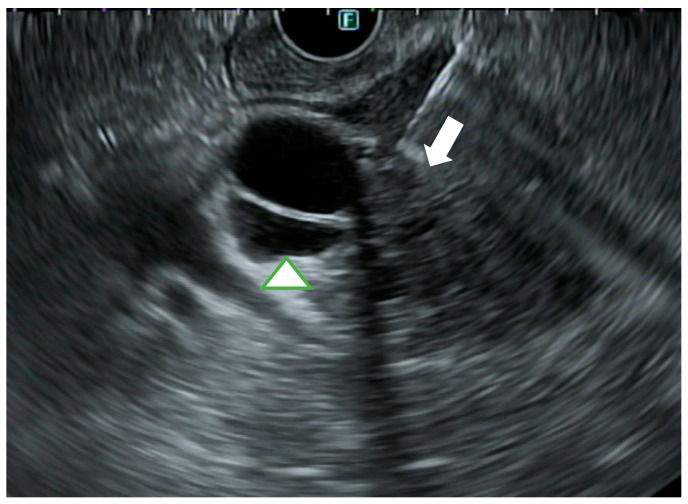
FNB of a suspicious extrahepatic cholangiocarcinoma (white arrow) with cystic duct involvement (arrowhead).

EUS’s role in defining biliopancreatic neoplasms and bile duct strictures has constantly increased over the last few years. EUS can provide a high-quality real-time image of the biliopancreatic system and allows for the obtention samples for cytology and histology through fine-needle aspiration (FNA) and fine-needle biopsy (FNB, Figure 3). Some studies demonstrated EUS’s better sensitivity in detecting distal strictures over proximal ones, which is the reason why ERCP remains the preferred initial approach in patients with proximal strictures. A single-center observational study showed that in a population of patients affected by CCA, EUS detected it in all distal tumors (100%) and in 83% of proximal tumors, with better performance of EUS over CT scans in tumor detection (94% vs. 30%, respectively) [9]. In particular, the yield of EUS with FNA in distal biliary strictures is excellent, with a reported sensitivity of 84% to 91% and specificity of 71% to 100%. The diagnostic yield of EUS with FNA in proximal biliary strictures is more variable. The reported sensitivity and specificity in this setting range from 45% to 89% and 79% to 100%, respectively [10]. Another interesting point is that combining EUS + ERCP further increases diagnostic chances: a multicenter study including 263 patients with suspected malignant biliary obstruction who underwent same-session EUS and ERCP concluded that overall diagnostic sensitivity and accuracy were 73.6% and 76.1% for EUS-FNA, 56.5% and 60.5% for ERCP, and 85.8% and 87.1% for the EUS/ERCP combination [11]. A recent meta-analysis involving 497 patients focused on same-session EUS and ERCP for tissue diagnosis compared to each method alone: the sensitivity, specificity, and accuracy for EUS-FNA alone were, respectively, 76%, 100%, and 94.5%; for ERCP-based tissue sampling, these variables were 58%, 98%, and 78.1%. For the same-session combined techniques, the sensitivity, specificity, and accuracy increased to 86%, 98%, and 96.5%, respectively. Interestingly, the analysis of each method in detecting pancreatic and biliary etiologies showed that EUS-FNA was superior to ERCP-based tissue sampling for pancreatic lesions, whereas for biliary ones, both methods had similar sensitivities [12]. A controversial consideration is the risk of needle tract seeding leading to metastases. Heimbach et al. [13] have demonstrated peritoneal metastases in 83% (5/6) of patients who underwent the FNA of unresectable hilar cholangiocarcinoma. Unfortunately, they did not distinguish between the percutaneous and EUS approaches. A few studies have examined the risk of seeding among distal common bile duct malignant strictures but with conflicting results [14,15,16,17,18,19]. Overall, based on the evidence to date, EUS with FNA/FNB is not recommended for proximal biliary strictures (in this case, lymphadenopathies can be a good FNA/FNB target to improve diagnostic chances), whereas the risk associated with distal biliary strictures remains unclear [20].

### 3.3. Intraductal Ultrasound (Miniprobe Endoscopic Ultrasound)

Intraductal ultrasound (IDUS) is performed during ERCP by inserting a small (2.4 mm in diameter), high-frequency (usually 20 MHz) catheter probe over a wire via a monorail design into the bile duct. The transducer rotates at the end of the probe and generates 360° images in a plane perpendicular to the catheter axis (similar to the radial echoendoscope). Malignant strictures tend to disrupt the normal three-layer appearance of the bile duct, have an asymmetric echo-poor mass with irregular margins, and infiltrate the surrounding tissue. Benign strictures often show the preservation of the bile duct wall layers and have a symmetric echo-rich mass with smooth margins [21]. The yield of IDUS in predicting malignant biliary stricture is good, with a sensitivity of 83% to 91% and a specificity of 80% to 93%. IDUS may be the most clinically useful tool when no mass is seen on cross-sectional or EUS imaging, or when EUS with FNA is negative and there is a suspicion of malignancy, thus increasing ERCP with brush sensitivity [10].

### 3.4. Cholangioscopy

**Figure 4:**
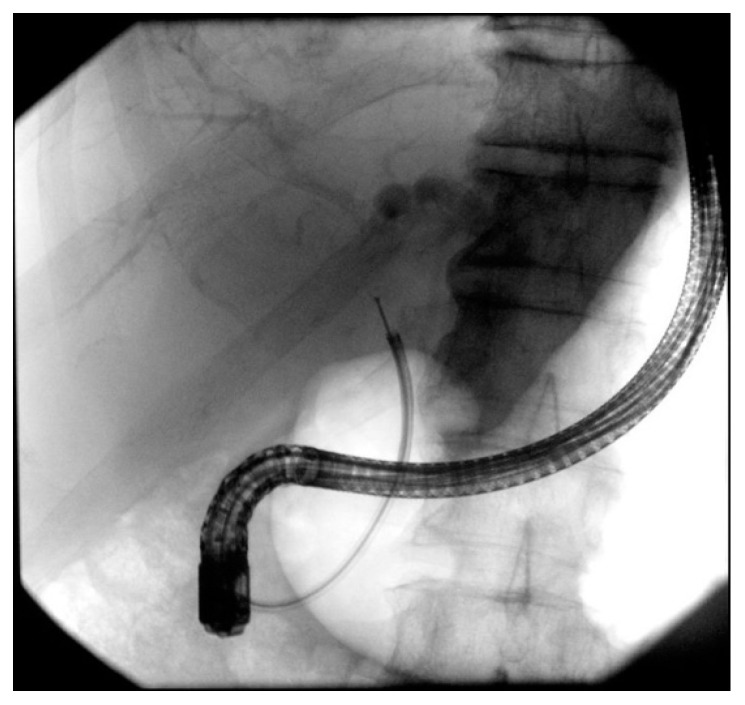
Spyglass device with spybite forceps during cholangioscopy.

**Figure 5:**
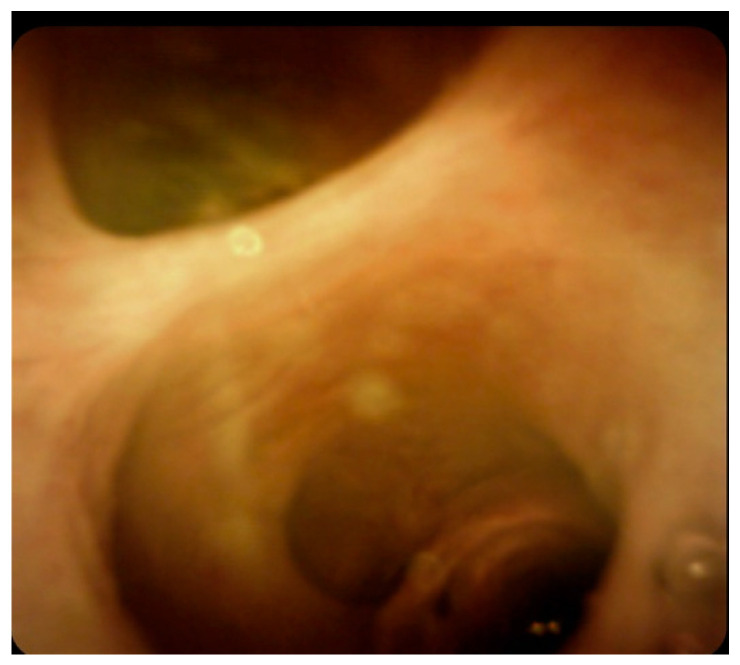
Regular hepatic confluence appearance during cholangioscopy.

**Figure 6:**
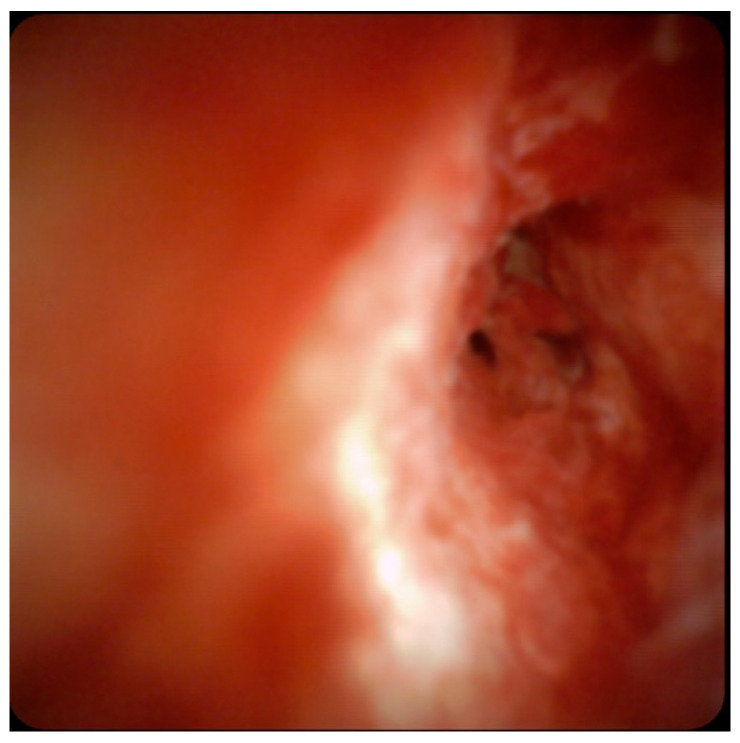
Hilar cholangiocarcinoma stricture appearance during cholangioscopy.

**Scheme 1:**
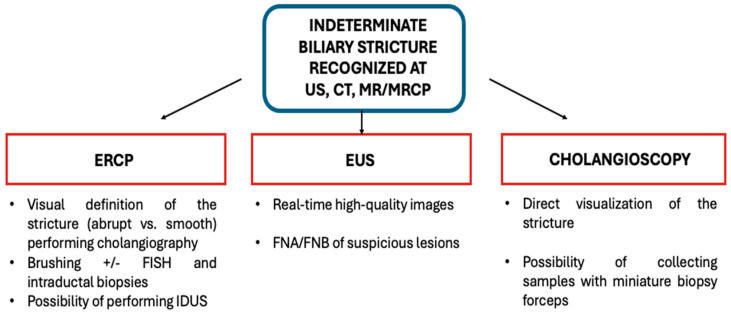
Schematic diagnostic approaches available to manage indeterminate biliary strictures.

Cholangioscopy is an endoscopic technique that allows direct bile duct lesions visualization with the opportunity to take samples with specific miniature biopsy forceps (Figure 4). During cholangioscopy, the visual impression of the bile ducts constitutes an integral part of the study (Figure 5). Signs of malignancy are, for example, tortuous dilated vessels, infiltrative strictures, polypoid or vegetative lesions, and easily bleeding ones (Figure 6). Benign features are villous or polypoid patterns without vascularity, fibrous or congestive patterns with inflammation signs, or a smooth epithelium [22]. In a recent meta-analysis including 283 procedures, the overall pooled sensitivity and specificity of direct single-operator cholangioscopy in the visual interpretation of biliary malignancies was 94% and 95%, respectively [23]. Direct biopsy collection through cholangioscopy is another advantage of this procedure: a meta-analysis revealed a pooled sensitivity of 71.9% of cholangioscopy-directed biopsies in diagnosing malignancy and a pooled specificity of 99.1% [24]. Despite these advantages, cholangioscopy has some limitations: it is not universally accessible and is an operator-dependent technique, with high costs and associations with serious and frequent adverse events due to bile duct direct access (such as cholangitis, pancreatitis, and bleeding). We summarize below the diagnostic tools available in biliary stricture settings (Scheme 1).

### 3.5. Confocal Laser Endomicroscopy

Probe-based confocal laser endomicroscopy (CLE) can provide in vivo imaging of biliary lesions during ERCP and additional diagnostic information [25,26]. A contrast agent is typically injected intravenously, and the biliary tissue is imaged with a CLE probe. This technique results in specific patterns that correlate with histology and help differentiate between malignant and normal biliary mucosa [27]. Miami classification is used to differentiate probe-based CLE visual findings as benign or malignant strictures. The presence of thick dark bands (>40 m), thick white bands (>20 m), dark clumps, epithelial strictures, villous glands, and contrast leakage can distinguish the nature of strictures [28]. Paris classification is a newer classification that includes additional features like vascular congestion, dark glandular patterns, interglandular spaces, and a thickened reticular structure [25]. CLE can be a promising tool but currently, its use is limited due to its cost and the lack of interobserver agreement [29].

### 3.6. Other Diagnostic Approaches

Vascular endothelial growth factor level in bile, fluorescence in situ hybridization for brush cytology, and methionyl-tRNA synthetase 1 staining for brush cytology are reported to be promising in pilot studies, but we need stronger evidence about their clinical value [29,30,31].

## 4. Management and Therapeutic Approach

When we suspect a biliary stricture, whatever its etiology, it is crucial to re-establish regular biliary flow into the duodenum. Restoring lower or normal bilirubin levels relieves patient symptoms such as itching due to jaundice and, in oncological patients, allows for the starting of chemotherapies. Indeterminate biliary strictures should be considered malignant unless proven otherwise. In patients with an extrahepatic or hilar indeterminate stricture, with high-serum bilirubin levels or with cholangitis or intractable itching, we need appropriate biliary drainage. Endoscopic treatment, particularly ERCP with stent placement, remains the first-line treatment in this setting. EUS-guided biliary drainage could be a good option in cases of failed ERCP. The percutaneous approach could still be an acceptable alternative in all cases of endoscopic approach failure or ineffectiveness.

### 4.1. ERCP in Distal Strictures

In extrahepatic strictures amenable to surgical approach, generally, the current guidelines recommend against routine preoperative biliary drainage (PBD), which should be reserved for symptomatic patients (cholangitis, itching, etc.) or for those who will undergo delayed surgery or neoadjuvant chemotherapy. Many meta-analyses and literature reviews have focused on the benefits and adverse events of preoperative biliary drainage: one of them [32] showed that preoperative biliary drainage increases wound and bile infection rates, but without affecting mortality and morbidity. A 2016 meta-analysis comparing mortality and adverse events in patients treated with PBD vs. direct surgery (DS) showed that in patients with malignant biliary jaundice requiring surgery, the PBD group had significantly fewer major adverse effects than the DS group [33]. Conversely, another study evaluating the incidence of overall complications and wound infection in these two groups found that there was a greater increase in overall complications and wound infections in the PBD group than in the DS group [34]. However, when the choice of biliary drainage in a preoperative setting is made, the current guidelines [35,36] recommend a self-expandable metal stent (SEMS) placement over a plastic one. It has been demonstrated by many meta-analyses that placing SEMS over plastic stents reduces the risk of stent dysfunction/cholangitis, requires fewer reinterventions, and ensures better patency periods and longer patient survival [37,38,39]. Then, when we have to choose between covered or uncovered SEMS, we must consider that the current guidelines [35] recommend against uncovered SEMSs (uSEMSs) in strictures of unconfirmed etiology. uSEMSs indeed have a poor long-term patency in obstructions that eventually turn out to be benign, because even in these ones, there could be tissue hyperplasia and ingrowth or sludge formation: complete stent embedding could lead to recurrent occlusion or cholangitis. Moreover, they are difficult to remove even with a “stent-in-stent” technique, which could be related to adverse events.

### 4.2. ERCP in Perihilar Strictures

Perihilar obstruction management is more difficult than distal ones and often requires a multidisciplinary approach in a referral center. Even in these kinds of strictures, the role of preoperative drainage in surgical candidates is debated due to increased postoperative morbidities, such as a greater infection rate or more adverse events. For these reasons, also in this setting, PBD is reserved for selected cases (delayed surgery, high serum bilirubin, and itching) and is not a routine suggested technique. When performed in the preoperative setting, the first choice is PBD by means of plastic stents with a side-by-side (SBS) bilateral stenting technique. Conversely, in palliative cases, many meta-analyses have indicated that SEMSs are superior to plastic ones in terms of prolonged overall survival, a lower rate of cholangitis, higher clinical success, and longer stent patency [40,41,42]. Particularly, the current guidelines [35] suggest uSEMSs in these cases. In uncertain-etiology strictures, multiple plastic stents are the best choice. Under fluoroscopic guidance, we can obtain appropriate liver drainage by a SBS stenting approach. In these cases, especially if the stricture is tight, it is often easier to place two 7 Fr stents initially to gradually dilate the bile duct and then replace them later with 10 Fr stents [43]. It is crucial to remember that the technical goal in hilar drainage has to be evaluated in volumetric sectorial terms and not in bilateral or unilateral terms: the final target is to promote the drainage of as much of the remaining liver as possible.

### 4.3. EUS-Guided Biliary Drainage (EUS-BD)

In the case of failed ERCP (papillary or duodenal infiltration, for example) an EUS-guided approach could be the solution. EUS could be helpful in obtaining anterograde access to the bile duct when papilla cannulation is not possible through a rendezvous technique, which consists of puncturing a bile duct with an access needle and introducing from there a guidewire through the papilla. In other cases, EUS is used to create a biliary–enteric fistula through a LAMS (lumen-apposing metal stent) that could provide a drainage proximal to the stricture. Examples of biliary–enteric EUS-guided fistulas are choledochoduodenostomy or hepaticogastrostomy, which are selected depending on several factors (such as stricture position or vascular interposition). Many meta-analyses have investigated EUS-BD’s role and its technical success. In 2017, Moole et al. [44] compared EUS-BD to the percutaneous transhepatic approach (PTBD), describing a successful biliary drainage with EUS in 90.91% of the pooled patient population with a pooled odds ratio of 3.06 for successful biliary drainage in the EUS-PD versus PTBD groups. Another meta-analysis evaluating the intrahepatic and extrahepatic approaches in EUS-BD showed a weighted pooled rate of 94% for technical success. Notably, in the meta-regression model, distal CBD strictures and transpapillary drainage were associated with higher technical success while the intrahepatic access route was linked to a higher adverse event rate [45]. Finally, another comprehensive literature review about the overall efficacy and safety of EUS-BD reported a pooled technical success rate of 91.5% and a clinical success rate of 87%, respectively [46].

### 4.4. Percutaneous Transhepatic Biliary Drainage (PTBD)

Percutaneous transhepatic biliary drainage consists of puncturing a bile duct under fluoroscopic or ultrasound guidance and obtaining a cholangiography by injecting contrast. Then, a guidewire is inserted through the needle to gain access to the biliary tree. To establish biliary drainage, we need to insert a catheter over the guidewire across the obstruction, providing an external drainage route transcutaneously. Today, this approach is not the first choice, especially if we have other options and if the stricture’s nature is not established. However, it may be a mandatory choice if endoscopic treatment is contraindicated, is not feasible, or has failed. Some recent meta-analyses [47,48], comparing endoscopic biliary drainage and percutaneous transhepatic biliary drainage, showed no difference in terms of technical success. One study, comparing specifically EUS-BD to PTBD, interestingly indicated that EUS-BD was associated with lower reintervention rates and lower odds of adverse events. Even the total treatment costs and the hospital stay were significantly lower in the EUS-BD group than in the PTBD group [49]. Thus, PTBD is reserved exclusively for endoscopic approach failure or non-feasibility.

## 5. Conclusions and Future Directions

Indeterminate biliary strictures still represent a diagnostic and therapeutic challenge for endoscopists. What we know is that the approach must be a multidisciplinary one, tailored to a single patient, and based on his or her personal history, preferably in a referral center. Often, a single diagnostic technique is not adequate to secure a proper diagnosis. What is determined is, therefore, that the key is to integrate different techniques in order to increase diagnostic chances and that a single-tissue sampling method is not always quite enough. Our future goal is to improve our diagnostic skills to minimize the total number of uncertain-etiology strictures to “determinate the indeterminate”.

## Figures and Tables

**Table 1:** Main causes of malignant and benign biliary strictures.

Malignant	Benign
**Pancreatic cancer**	*Inflammatory:* chronic pancreatitis, PSC, IgG4-related cholangitis/pancreatitis
**Cholangiocarcinoma**	*Iatrogenic:* cholecystectomy, liver transplant, chemotherapy, radiation
**Gallbladder malignancy**	*Vascular:* ischemic biliary injury, portal hypertensive biliopathy, vasculitis
**Hepatocellular carcinoma**	*Infectious:* recurrent pyogenic cholangitis, HIV cholangiopathy, parasitic infections
**Ampullary cancer**	*Traumatic*
**Lymphoma**	Mirizzi syndrome
**Metastasis**	*Other:* sarcoidosis, eosinophilic cholangitis, mastocytosis
